# Rapid Assembly of Multiple-Exon cDNA Directly from Genomic DNA

**DOI:** 10.1371/journal.pone.0001179

**Published:** 2007-11-14

**Authors:** Xiaoping An, Jun Lu, Jian-dong Huang, Baozhong Zhang, Dabin Liu, Xin Zhang, Jinhui Chen, Yusen Zhou, Yigang Tong

**Affiliations:** 1 State Key Laboratory of Pathogen and Biosecurity, Beijing Institute of Microbiology and Epidemiology, Beijing, China; 2 Beijing YouAn Hospital, Capital Medical University, Beijing, China; 3 Department of Biochemistry, The University of Hong Kong, Hong Kong Special Administrative Region (SAR), China; National Cancer Institute at Frederick, United States of America

## Abstract

**Background:**

Polymerase chain reaction (PCR) is extensively applied in gene cloning. But due to the existence of introns, low copy number of particular genes and high complexity of the eukaryotic genome, it is usually impossible to amplify and clone a gene as a full-length sequence directly from the genome by ordinary PCR based techniques. Cloning of cDNA instead of genomic DNA involves multiple steps: harvest of tissues that express the gene of interest, RNA isolation, cDNA synthesis (reverse transcription), and PCR amplification. To simplify the cloning procedures and avoid the problems caused by ubiquitously distributed durable RNases, we have developed a novel strategy allowing the cloning of any cDNA or open reading frame (ORF) with wild type sequence in any spliced form from a single genomic DNA preparation.

**Methodology:**

Our “Genomic DNA Splicing” technique contains the following steps: first, all exons of the gene are amplified from a genomic DNA preparation, using software-optimized, highly efficient primers residing in flanking introns. Next, the tissue-specific exon sequences are assembled into one full-length sequence by overlapping PCR with deliberately designed primers located at the splicing sites. Finally, software-optimized outmost primers are exploited for efficient amplification of the assembled full-length products.

**Conclusions:**

The “Genomic DNA Splicing” protocol avoids RNA preparation and reverse transcription steps, and the entire assembly process can be finished within hours. Since genomic DNA is more stable than RNA, it may be a more practical cloning strategy for many genes, especially the ones that are very large and difficult to generate a full length cDNA using oligo-dT primed reverse transcription. With this technique, we successfully cloned the full-length wild type coding sequence of human polymeric immunoglobulin receptor, which is 2295 bp in length and composed of 10 exons.

## Introduction

Gene cloning is one of the most frequently used technologies in a molecular biology laboratory. To study a particular gene, the first step is usually to clone and express it. However, most of the eukaryotic genes are interrupted by intervening sequences (introns), which make the gene of interest very large. Manipulation of the large genomic DNA is tedious and problematic due to size capacity of cloning vectors and multiple restriction endonucleases which make it difficult to find appropriate enzymes for subcloning. To circumvent these difficulties, the cDNA is often used instead of its large genomic counterpart. But cDNA cloning is usually troublesome, which involves mRNA preparation and reverse transcription, and thus requires RNA extraction kits and reverse transcription kits. For a particular cDNA cloning, a specific tissue with a relatively high level expression is often needed for the mRNA or total RNA extraction. Some of these tissues are quite rare and difficult to obtain, and some of the genes only have a very low level expression. Furthermore, RNases are ubiquitously expressed in tissues and RNase products are commonly used in molecular biology protocols (such as plasmid DNA preparation). Since RNases are very stable and difficult to remove completely, extra care must be taken when working with RNA. Lastly, RNAs are unstable polynucleotides and thus present their own problems in handling and storage.

As the genomic DNA sequence and annotation databases skyrocketed in recent years, it has become easier to obtain information on virtually any gene and the corresponding protein sequence. With the advanced technology and low cost of oligonucleotide synthesis, *de novo* gene synthesis is becoming a common gene cloning option. Several gene synthesis strategies have been developed over the past three decades, including oligonucleotide ligation [1]–[3], the *Fok*I method of gene synthesis[4], [5], self-priming PCR [6]–[8], dual asymmetric PCR[9], combined dual asymmetric PCR and overlap extension PCR [10], assembly PCR [11]–[13] and successive PCR [14]–[16]. Assembly PCR was originally adopted in DNA shuffling for *in vitro* evolution of DNA molecules [17], [18]. With assembly PCR or successive PCR, any DNA sequence can be easily obtained *de novo* by assembling synthetic oligonucleotides. This technique makes DNA cloning very simple and is applied widely and extensively in molecular biology studies. To help design the assembly primers, as well as to optimize codon usage for various expression systems, some dedicated computer software [19] and internet online tools [12], [20] have been developed.

Here we report a novel PCR-based *in vitro* genomic DNA splicing strategy (designated as “PCR mediated Genomic DNA Splicing” or GDS strategy) for cloning of any eukaryotic cDNA or coding sequence from a genomic DNA preparation. This genomic DNA preparation serves as a universal PCR template and can be prepared from any tissue including peripheral blood cells. As for now, several human and mouse cDNAs have been cloned in our laboratories from a human genomic DNA template and a mouse genomic DNA template respectively, and here we take the multiple-exon human polymeric immunoglobulin receptor (*PIGR*) gene as an example to illustrate this novel cDNA cloning strategy.

## Materials and Methods

### Chemicals, enzymes and stains

*Taq* DNA polymerase, T4 DNA ligase, DL2000 DNA marker, Blood Genome DNA Extraction Kit, and restriction endonucleases were purchased from Takara Biotech Co., Ltd. (Dalian, China). Phusion high-fidelity DNA polymerase and T7 endonuclease I were from New England Biolabs, Inc. (Ipswich, MA). IPTG, X-Gal, and other chemicals were from Sigma-Aldrich, Inc. (St Louis, MO). Oligonucleotides were synthesized and purified by polyacrylamide gel electrophoresis by AuGCT Biotech Co., Ltd. (Beijing, China). TA cloning vector (pGEM®-T easy vector) was purchased from Promega Biotech Co., Ltd (Madison, WI). DH5α competent cells were from Tiangen Biotech Co., Ltd (Beijing, China). Glass milk DNA purification kits were from BioDev-Tech Co., Ltd (Beijing, China).

### Experimental design

**Figure 1:**
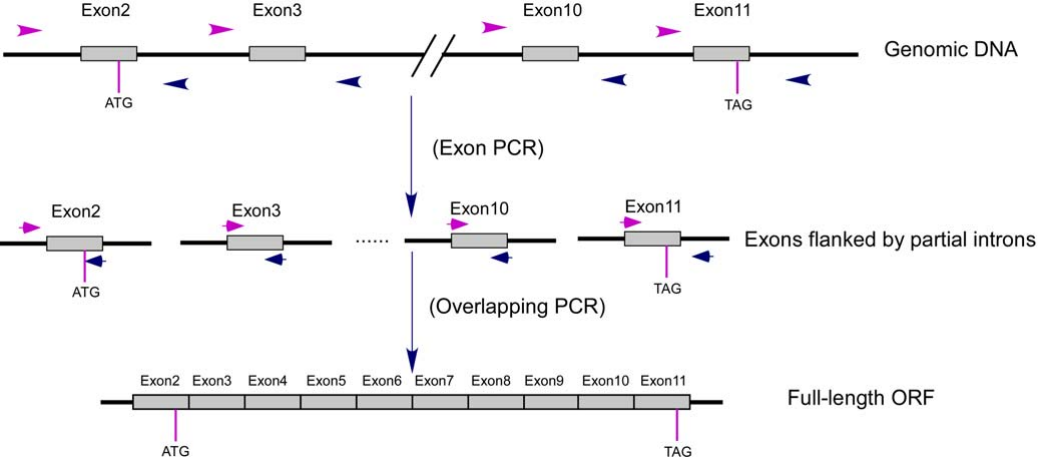
Schematic diagram of PCR mediated Genomic DNA Splicing (GDS) strategy. Individual exons are amplified in the first PCR (exon PCR) following standard PCR protocol with software-optimized primers. A second PCR (overlapping PCR) is then carried out to join the exons and produce the full-length coding sequence. ATG and TAG indicate the start and stop codons, respectively.

The principle of this strategy is composed of three steps (Figure 1): i) Amplification of all the exons of the desired gene with optimized primers within flanking introns. ii) Joining of exons by overlapping PCR. iii) Amplification of full-length products. The rationale of this strategy is as follows: All the exon sequences of a gene exist in the genome. But due to the low copy number (usually only one copy per genome) of every exon and the extremely high complexity of the genome, it is usually impossible to successfully amplify the full-length cDNA sequence by overlapping primers located at the precise splicing sites which are not possible for software optimization. However, it is always easy to obtain any single exon (usually together with some flanking intron sequences) from a genomic DNA preparation by PCR with software-optimized, highly efficient primers located in flanking introns. Then, by assembling the above exons with deliberately designed overlapping primers, it is easy to amplify the full-length cDNA or ORF of interest with software-optimized, highly efficient outmost primers.

### Preparation of universal genomic DNA template

A two hundred microliter whole blood sample was collected from a healthy donor and the genomic DNA was extracted with Blood Genome DNA Extraction Kit (Takara) according to the manufacturer's instructions. The purified DNA was dissolved in 50 µl TE buffer (10 mM Tris•Cl, pH 8.0, 1 mM EDTA) and its concentration was determined by an ultraviolet spectrophotometer (Milton Roy Spectronic 601, Bio-Rad Laboratories, Inc., Richmond, CA).

### Exon amplification and assembly

Exon amplification was performed in individual tubes on a GeneAmp PCR system 2400 (Perkin Elmer, Foster City, CA). For each exon, a standard PCR was carried out with 25 µl total reaction mixture containing 50 mM KCl, 10 mM Tris-Cl, 1.5 mM MgCl_2_, 10 ng genomic DNA, 0.2 mM dNTP, 1 µM of each forward and reverse primer, and 0.5 unit Taq polymerase. The PCR parameters were as follows: pre-denaturation at 94°C for 3 minutes, followed by 35 cycles of denaturation at 94°C for 30 seconds, annealing at 55°C for 30 seconds, and polymerization at 72°C for 60 seconds, and a final incubation for 5 min at 72°C. The PCR products were pooled and used as a template in the subsequent overlapping PCR. In this second, overlapping PCR reaction, the overlapping PCR primer pairs of all the exons and the outmost primers (0.1 µM each) were pooled and used in a 25 µl reaction mixture containing 2 µl of the pooled template and the other components (see above). After 20 cycles of amplification (denaturation at 94°C for 30 seconds, annealing at 55°C for 30 seconds, and polymerization at 72°C for 120 seconds), 2 µl of the product was transferred into the final amplification tube with a total volume of 50 µl containing the other components as above and with the outmost primers to amplify the full-length ORF sequence following a standard PCR protocol. The PCR products were subjected to 1% agarose gel electrophoresis and the gel containing full-length DNA was excised and the DNA was purified with a glass milk kit (BioDev-Tech).

### PCR with the Phusion high-fidelity DNA polymerase

When the Phusion high-fidelity DNA polymerase was used in place of Taq polymerase, the thermocycle parameters were adjusted as follows: pre-denaturation at 98°C for 30 seconds, denaturation at 98°C for 15 seconds, and annealing at 60°C for 30 seconds, with other parameters unchanged. To add an A base at the 3′ terminus, the full-length PCR products were purified by extraction with equal volume phenol-chloroform-isoamyl alcohol (25:24:1) and precipitation with three volume ethanol. The DNA pellet was dissolved in 50 µl standard PCR reaction mixture (components see above, without primers) containing 1 unit Taq DNA polymerase. After incubation at 72°C for 20 minutes, the DNA was separated by 1% agarose gel electrophoresis and the full-length DNA was purified from the gel with a glass milk kit (BioDev-Tech).

### T7 endonuclease I treatment of PCR products

The 3′ A tailed DNA products amplified by the Phusion high-fidelity DNA polymerase from two PCR reactions were dissolved in 15 µl distilled water and subjected to denaturation at 98°C for 1 minute, and then a stepwise annealing procedure was carried out as follows: 2 minutes at 85°C, 3 minutes at 75°C, 3 minutes at 65°C, 3 minutes at 37°C, followed by 3 minutes at room temperature. The T7 endonuclease I treatment was performed in a 20 µl solution containing the above DNA and 15 units of T7 endonuclease I as well as 1×T7 endonuclease I buffer. The mixture was incubated at 37°C for 1 hour to allow the mismatched molecules to be cleaved by the T7 endonuclease I.

### Cloning and sequencing

The PCR products generated by Taq DNA polymerase or 3′ A tailed PCR products generated by the Phusion high-fidelity DNA polymerase were ligated with pGEM®-T easy vector (Promega). The ligation mixture was transformed into DH5α competent cells (Tiangen) and white colonies were picked and insertion was analyzed by PCR and restriction enzyme cleavage. Positive clones were subjected to DNA sequencing using an automated DNA sequencer ABI377 (Perkin Elmer).

## Results

### Exon Primer Design

**Figure 2:**
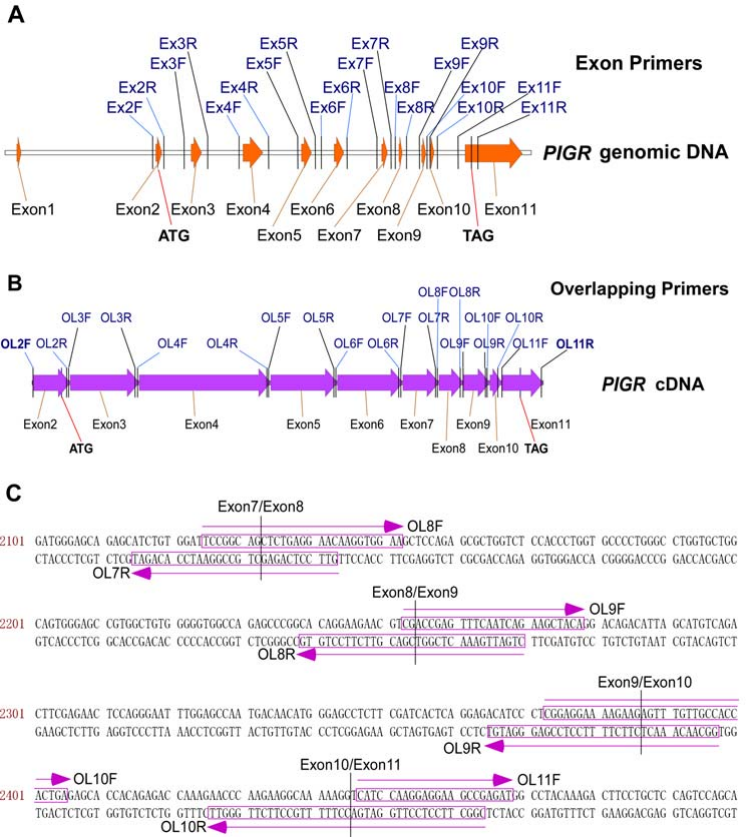
Primer design for *PIGR* ORF cloning by GDS strategy. A. Exon primers for exon PCRs. All exons are represented by solid arrows and are flanked by optimized forward and reverse primers located in introns. The start codon resides in exon 2 and stop codon in exon 11. Exon 1 does not contain a coding sequence and was not amplified. For exon 11, only the region containing the coding sequence was amplified. B. Overlapping primers for overlapping PCR. C. Alignment of the overlapping primers with the cDNA sequence (only a portion is shown). Primers are boxed and their orientations are indicated by parallel arrows. Exon-exon junctions are marked by vertical lines.

An online program EasyExonPrimer [21] (http://129.43.22.27/∼primer/EasyExonPrimer.html), which is a free Internet service hosted by NCI, was used to design the exon amplification primers for the first round PCR. The human *PIGR* mRNA in NCBI GenBank database was retrieved, and the GenBank accession number as NM_002644 was obtained and submitted using this program. Default parameters were used for primer selection, i.e.: an optimal primer size of 22nt (18∼25 nt); an optimal Tm of 60°C (45°C∼65°C); an optimal primer GC percentage of 45 (30%∼60%); an optimal product Tm of 60°C (45°C∼65°C); a maximal self-complementarity of 8bp; and a maximal 3′ self-complementarity of 3bp. Exon 1 does not contain any coding sequence, and is excluded from amplification. Since exon 11 is longer than 1000 bp, which may make it difficult to be amplified, this exon sequence was broken into several smaller fragments for primer design. Only the first fragment of exon 11 contains coding sequence, thus only the primer pairs for this fragment were synthesized along with the primers of exon 2 though 10. The relative primer positions are represented in Figure 2A and the primer sequences are listed in Table 1.

**Table 1:** Primer sequences.

	Exon Primers (5′----3′)	
	Forward	Reverse
Exon2	Ex2F GTTATGGAAAAAGTGGAGGCAC	Ex2R TCTTCTCTGTATGACTCTTCCTCAA
Exon3	Ex3F CCCTTGTCCTTGAAATGGTTATC	Ex3R GTTATAGGCCACCAAGTCCTAGAAG
Exon4	Ex4F GAAGGAGGGCATTCCAGGTTAT	Ex4R AGAGTAATTAGGAGCTGCCATCAG
Exon5	Ex5F AAGGTGATGCGTGTAAACTTCA	Ex5R AATGTGACCTCTGGATAGGTGG
Exon6	Ex6F TCCATGTGTGAAGATCTATGGTCTA	Ex6R ACTCTCCACTTTCAGCCTCTTACAG
Exon7	Ex7F CATCACAGCTGTTTCTAATGTCATC	Ex7R CCTCCCACATATAAGCAGAGGTAGT
Exon8	Ex8F TCTTAGAAGAACAGAGCTAGGGAGC	Ex8R CTTTCTCGGGATCCTTTGTTTTAG
Exon9	Ex9F CATAGTCAGAGCTGCACCACCT	Ex9R GCAACAAACTCTGTGTGAAGAAAG
Exon10	Ex10F GAAAAGAAGGTATGACCCTCACTC	Ex10R GCATCCCACAACCAGTAAGATATAG
Exon11	Ex11F GTCAGAAGGAGGTAGTGAGACAGAA	Ex11R CTCTGAGGACAGTAGGAAAAACCTA
	Overlapping Primers (5′----3′)	
	Forward	Reverse
Exon2	**OL2F** CAAAGCCAAATAGATGTC	OL2R CGTGGAGATGGCTGGGAAGACCGCCAGCA
Exon3	OL3F CTTCCCAGCCATCTCCACGAAGAGTCC CATA	OL3R AGGAGCCCAGGACCCTGGCTGACCTCCAGGCTGA
Exon4	OL4F CAGCCAGGGTCCTGGGCTCCTAAATGA	OL4R CGTGGACTCCTCATTGACGAAGAGTTG
Exon5	OL5F CGTCAATGAGGAGTCCACGATTCCCCG CA	OL5R TTGGTTCTCCTTCGATAATCTTGATCT
Exon6	OL6F TATCGAAGGAGAACCAAACCTCAAGGTA	OL6R CGCGGGACCCCGCTGCCTTCCTCTCTTCAA
Exon7	OL7F AAGAGAGGAAGGCAGCGGGGTCC CGCGATGTCAGCCTA	OL7R GTTCCTCAGAGCTGCCGGAATCCACAGAT
Exon8	OL8F TCCGGCAGCTCTGAGGAACAAGGTGGAA	OL8R CTGATTGAAACTCGGTCGACGTTCTTCCTGTG
Exon9	OL9F CGACCGAGTTTCAATCAGAAGCTACA	OL9R GGCAACAAACTCTTCTTTTCCTCCGAGGGATGT
Exon10	OL10F CGGAGGAAAAGAAGAGTTTGTTGCCA CCACTGA	OL10R CGGCTTCCTCCTTGGATGACCTTTTTGCCT TCTTGGGTT
Exon11	OL11F CATCCAAGGAGGAAGCCGAGAT	**OL11R** CCTAGGCAGGTGTTAGAG

### Overlapping primer and outmost primer design

Overlapping primers were manually designed at the exon-exon joining sites. To avoid a mismatch caused by an additional A base at the 3′ end produced by Taq polymerase, all the overlapping primers start after a T base. Other considerations for the overlapping primer design were no 3′ self-pairing and low 3′ G/C content. The length of the overlapping primers varied from 27 bp to 39 bp, with a minimal overlapping region of 15 bases. The outmost primers (OL2F and OL11R) for the full-length ORF amplification do not contain any overlapping sequence and were selected using computer software (Oligo version 6.71, Molecular Biology Insights, Inc., Cascade, CO). The positions of the overlapping primers and the outmost primers are illustrated in Figure 2B and their sequences are shown in table 1. Figure 2C shows alignment of the overlapping primers and the outmost primers with the *PIGR* coding sequence.

### Amplification of *PIGR* coding exons by standard PCR

**Figure 3:**
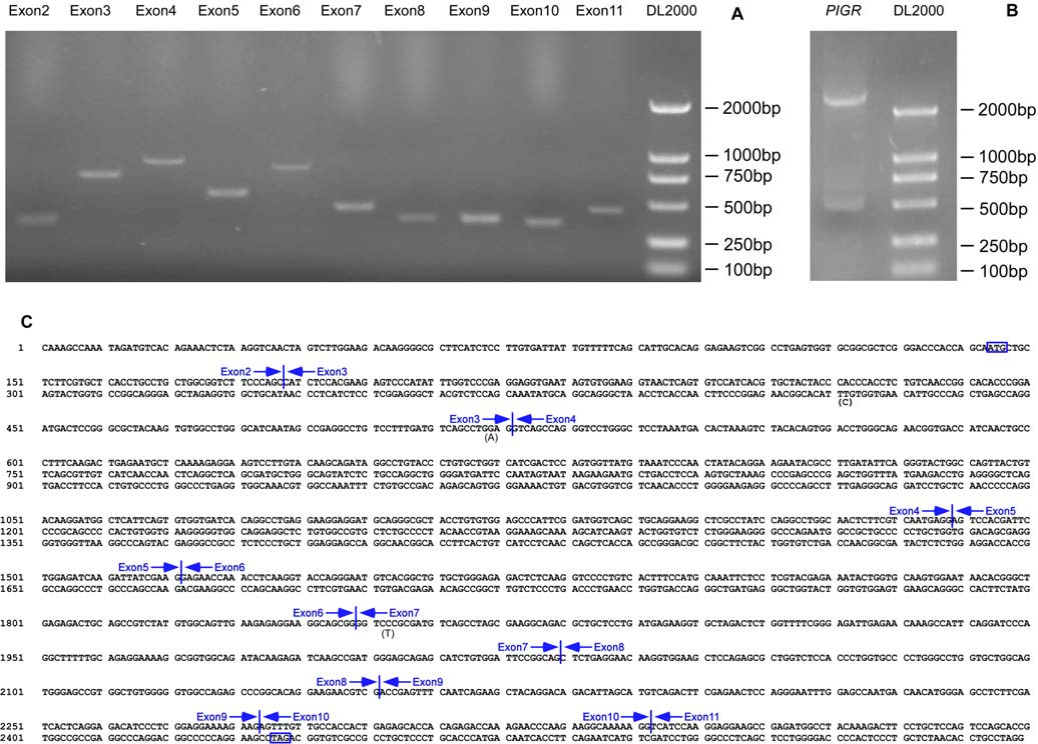
Amplification, assembly and cloning of *PIGR* coding sequence by GDS strategy. A. Amplification of *PIGR* coding exons by standard PCR protocol with exon primers. The sizes of the amplified fragments of exon 2 through 11 are 400 bp, 795 bp, 989 bp, 599 bp, 855 bp, 486 bp, 395 bp, 387 bp, 369 bp, 461 bp, respectively. B. Amplification of full-length *PIGR* coding sequence. The size of the amplified full-length sequence is 2549 bp, including 2295 bp *PIGR* ORF and two short flanking sequences. C. Exon assembly of *PIGR* coding sequence. The start and stop codons are boxed. Exon-exon junctions are marked by vertical lines. The point mutations mentioned in the text are indicated by the mutated bases in parenthesis under the wild type ones. DL2000, DNA molecular weight marker (Takara).

A genomic DNA preparation was made from a blood sample of a healthy donor and was used as a universal exon PCR template. Since exon 1 and majority of exon 11 of *PIGR* gene do not contain any coding sequence, we only amplified exon 2 though 10 and the first fragment of exon 11, using a standard PCR protocol. In order to test the possibility of multiple exon assembly, we just used ordinary Taq DNA polymerase to perform the PCRs. Without any optimization of the PCR conditions, all of the exon PCRs were successful as demonstrated by 1% agarose gel electrophoresis (Figure 3A). The predicted length of exon 2 through exon 11 are 400 bp, 795 bp, 989 bp, 599 bp, 855 bp, 486 bp, 395 bp, 387 bp, 369 bp, 461 bp (fragment 1), respectively.

### Assembly of full-length *PIGR* ORF by overlapping PCR

The above exon PCR products were pooled without purification and used as templates for a second round overlapping PCR to assemble the full-length *PIGR* coding sequence. To amplify the full-length PCR product, the outmost primers OL2F and OL11R were used to perform another round standard PCR. Agarose gel electrophoresis analysis of the final PCR product showed that dominant amplification product was a ∼2500 bp band (2295 bp *PIGR* ORF plus two short flanking sequences) (Figure 3B). To simplify the full-length product amplification, we tried to combine the 20-cycle overlapping PCR and the final PCR by running only a single PCR step with normal amount of outmost primers and reduced amount of overlapping primers (each overlapping primer concentration equals one-fiftieth of the outmost primer concentration). We found this simplified protocol could amplify the full-length product, but with a reduced amount of final product.

### Sequence analysis of *PIGR* cDNA clones

We cloned the full-length *PIGR* coding sequence into pGEM®-T Easy Vector. Five clones were picked up for DNA sequencing analysis and the results showed that all 5 clones contained correctly joined full-length *PIGR* coding sequence (Figure 3C), and since we used the error-prone Taq DNA polymerase in the above PCRs, there were an average of 2.5 point mutations per clone (the mutation rate is about 0.1%). Of all these mutations, nearly half located within the overlapping primers. Since the overlapping primers only compose a small fraction (much less than 50%) of the whole sequence, it suggests that synthetic oligonucleotides have a relatively high error rate[10], [11]. One of the clones had only two silent mutations and thus encoded wild type human polymeric immunoglobulin receptor (Figure 3C).

### Cloning of wild type cDNA with a high-fidelity thermostable DNA polymerase

In many cases wild type DNA sequences are required. In order to get wild type cDNA sequence, we then used the high-fidelity thermostable DNA polymerase Phusion DNA polymerase (New England Biolabs) to amplify the target DNA sequence in place of error-prone Taq polymerase. We sequenced three clones derived from Phusion DNA polymerase amplification and found two of them had the wild type sequence. But the other clone still contained a point mutation (G/A transition) at the location of the overlapping primer Ex3R (the mutation rate is about 0.01%) (Figure 3C). To further improve the method to get rid of possible point mutations, we applied T7 endonuclease I (New England Biolabs) to pre-treat the annealed full length PCR products before ligation into pGEM®-T Easy Vector. Since T7 endonuclease I hydrolyze the loop formed by mismatch between the mutant strand and the wide strand, it can prevent the error PCR products from ligation with T vectors. After transformation of the above ligation mixture, three positive clones were subjected to DNA sequencing. The results showed that all the three clones contained correctly assembled, full-length wild type *PIGR* coding sequence.

Similarly, we have cloned full-length coding sequences of human immunoglobulin J polypeptide gene (*IGJ*, NM_144646, 480 bp), human high-mobility group box 1 gene (*HMGB1*, NM_002128, 612 bp) and mouse urokinase-type plasminogen activator gene (*uPA,* NM_008873, 1302 bp). These successful cloning experiments suggest that our “Genomic DNA Splicing” technique is reliable and practicable.

## Discussion

PCR-based *de novo* gene synthesis is now frequently used in molecular biology laboratories worldwide, with the free online tools to automate the primer design and, more importantly, to optimize the codon usage for efficient expression in intended organism, such as for *E.coli* or yeast expression [12], [20]. However, this gene synthesis strategy (known as “assembly PCR”) requires large amounts of chemically synthesized oligonucleotides, multiple rounds of PCRs, and often results in numerous unwanted point mutations due to the large number of synthesized bases and multiple rounds of PCRs[10], [11].

Here we reported a new gene synthesis strategy, “PCR mediated Genomic DNA Splicing” (GDS), which resembles “assembly PCR” in procedures but takes advantage of genomic DNA to avoid synthesis of large amount of oligonucleotides, thus is cost-effective and can reduce the unwanted mutations introduced by chemical synthesis of large numbers of relatively long oligonucleotides[10]. In the case of *PIGR* ORF (2549 bp) cloning, if the “assembly PCR” method had been used, we had to synthesize about 5000 bases, and all of them would contribute to the final sequence. When the GDS method was used, only 1058 bases were synthesized, and among them, only 549 bases (less than one-ninth of the “assembly PCR”) contributed to the final sequence. It has been reported that assembly PCR results in a point mutation rate of 0.1% even when the proof reading thermostable DNA polymerase Pfu was used [11]. In our previous assembly PCR experiment using Taq polymerase, which is error-prone, we found an average of 3 point mutations in a 720bp *de novo* synthesized sequence (the mutation rate was 0.4%) [22]. However when we used the Taq polymerase to perform GDS cloning, the mutation rate was only about 0.1%.

In order to enhance the fidelity of our method and get wild type sequences, we tried to apply a high-fidelity thermostable DNA polymerase (namely Phusion high-fidelity DNA polymerase) for the PCRs. Since this DNA polymerase possesses a 3′-5′ proof-reading endonuclease activity, its mutation rate is 50-fold lower than that of Taq DNA polymerase and 6-fold lower than that of *Pyrococcus furiosus* (Pfu) DNA polymerase (as claimed by the manufacturer). To further reduce the mutation rate, we employed the T7 endonuclease I to cleave the mismatched loop formed between the mutated sequence and the wild type sequence after the PCR products were denatured and then annealed. Our results showed that the mutation rate was reduced to about 0.01% when the Phusion high-fidelity DNA polymerase was used alone, and no point mutation was identified when the T7 endonuclease I was used as a combination with Phusion high-fidelity DNA polymerase. For the 2549 bp DNA sequence cloned in this report, wild type sequences were obtained in both experiments with the high-fidelity DNA polymerase.

One advantage of the cDNA cloning strategy described in this report is that neither RNA extraction nor reverse transcription is needed, and thus it saves the costs of RNA extraction kits and cDNA synthesis kits. The second advantage is that it is not necessary to handle any specific tissues for a particular gene and the expression level of the gene is not a problem. The third advantage is that high fidelity DNA sequence is obtained with high efficiency while the process is simple and easy. The entire assembly process takes only a few hours. We found that even if some of the first round PCRs were not very successful (PCR product bands were not visible in ethidium bromide stained agarose gel), it usually did not affect the final full-length DNA amplification. So it is usually not necessary to optimize PCR conditions even if one or more exon primer pairs do not work very well in the first round PCR.

In order to make the primer design easy, we searched the Internet for appropriate programs to automatically design exon amplification primers, and found a few useful applications, including Genomic Primers (http://www2.eur.nl/fgg/kgen/primer/Genomic_Primers.html), ELXR (http://ecom2.mwgdna.com/services/elxr/elxrdb_query.tcllocEBE) [23], ampExon (http://bioinformatics.well.ox.ac.uk/mst-bin/ampexon.pl), ExonPrimer (http://ihg.gsf.de/ihg/ExonPrimer.html), and EasyExonPrimer [21] (http://129.43.22.27/∼primer/EasyExonPrimer.html). We chose to use EasyExonPrimer because it is highly automatic, very easy to use and free for use. This online tool is written in PERL and is based on a common PCR primer design program Primer3. We found that this online tool produced very efficient primers for exon amplification. All of our exon primers are designed by this software and almost all of them have successfully amplified the target sequences, including the exons of human *PIGR*, *IGJ*, *HMGB1* and mouse *uPA* genes, without PCR condition optimization. To design the exon-exon junction primers (overlapping primers, or splicing primers), we could not find any appropriate program. We tried to use an online application ExPrimer [24], which is a web based computer program to design primers from exon-exon junctions of a specific gene transcript, but the output exon-exon junction primers need to be paired by other primers located in exons, and these primers are not suitable for overlapping PCR. So we just designed all of our overlapping primers manually.

As for our knowledge, this is the first report of a rapid and efficient method to clone wild type multiple-exon cDNA sequences directly from a single genomic DNA preparation. Lebedenko *et al* reported an *in vitro* exon ligation method, but it involved complicated steps including two-step PCR to amplify the exons, cloning of all exons separately, introduction of specific restriction enzymes and ligation of multiple restriction fragments[25]. A similar PCR-based exon ligation method was proposed by Vandenbroeck *et al*, which required multiple rounds of PCR and multiple ligation reactions of blunt-end PCR products and restriction fragments[26]. Chistiakov *et al* used a PCR procedure to assemble exons of human interleukin 1 gene, but the exons were obtained from cloned DNA and only 3 exons were assembled by two rounds overlapping PCR, with each round of PCR to join only two exons (exon 5 and 6 were joined first, and then exon 5+6 was joined with exon 7)[27].

Thus we have developed a time- and cost-effective method for multiple-exon cDNA or ORF cloning directly from genomic DNA, which does not require tissue handling, RNA preparation and reverse transcription, and efficiently produces wild type full-length cDNA or ORF sequences.
